# Comparative Evaluation of Advanced Chunking for Retrieval-Augmented Generation in Large Language Models for Clinical Decision Support

**DOI:** 10.3390/bioengineering12111194

**Published:** 2025-11-01

**Authors:** Cesar Abraham Gomez-Cabello, Srinivasagam Prabha, Syed Ali Haider, Ariana Genovese, Bernardo G. Collaco, Nadia G. Wood, Sanjay Bagaria, Antonio Jorge Forte

**Affiliations:** 1 Division of Plastic Surgery, Mayo Clinic, Jacksonville, FL 32224, USA; 2 Department of Radiology AI IT, Mayo Clinic, 200 First St. SW, Rochester, MN 55905, USA; 3 Division of Surgical Oncology, Mayo Clinic, 4500 San Pablo Road, Jacksonville, FL 32224, USA; 4 Department of AI and Informatics, Mayo Clinic, 4500 San Pablo Road, Jacksonville, FL 32224, USA; 5 Center for Digital Health, Mayo Clinic, 200 First St. SW, Rochester, MN 55905, USA

**Keywords:** advanced chunking, adaptive chunking, retrieval-augmented generation, large language models, clinical decision support systems, patient safety

## Abstract

Retrieval-augmented generation (RAG) quality depends on how source documents are segmented before indexing; fixed-length chunks can split concepts or add noise, reducing precision. We evaluated whether proposition, semantic, and adaptive chunking improve accuracy and relevance for safer clinical decision support. Using a curated domain knowledge base with Gemini 1.0 Pro, we built four otherwise identical RAG pipelines that differed only in the chunking strategy: adaptive length, proposition, semantic, and a fixed token-dependent baseline. Thirty common postoperative rhinoplasty questions were submitted to each pipeline. Outcomes included medical accuracy and clinical relevance (3-point Likert scale) and retrieval precision, recall, and F1; group differences were tested with ANOVA and Tukey post hoc analyses. Adaptive chunking achieved the highest accuracy—87% (Likert 2.37 ± 0.72) versus baseline 50% (1.63 ± 0.72; *p* = 0.001)—and the highest relevance (93%, 2.90 ± 0.40). Retrieval metrics were strongest with adaptive (precision 0.50, recall 0.88, F1 0.64) versus baseline (0.17, 0.40, 0.24). Proposition and semantic strategies improved all metrics relative to baseline, though less than adaptive. Aligning chunks to logical topic boundaries yielded more accurate, relevant answers without modifying the language model, offering a model-agnostic, data-source-neutral lever to enhance the safety and utility of LLM-based clinical decision support.

## 1. Introduction

The evolving landscape of evidence-based medical practices, coupled with the immense workload faced by physicians, contributes significantly to professional burnout and elevates the risk of clinical errors, including misdiagnoses and treatment inaccuracies [1]. In this challenging environment, integrating artificial intelligence (AI) offers a promising avenue for support. Machine Learning (ML) and Deep Learning (DL) algorithms, which enable computers to learn without explicit programming and refine predictions autonomously, are prone to becoming transformative forces in healthcare [2,3,4,5,6], especially now with the advances in computational power and electronic health records (EHRs) [7].

Large Language Models (LLMs), a subset of DL, have shown strong performance in diagnostic support, imaging interpretation, and administrative automation [8,9,10,11,12,13,14,15,16,17,18]. They can synthesize evolving medical evidence, achieving accuracy comparable to physicians [19] and reducing administrative burdens [20].

Nevertheless, traditional LLMs are not without limitations. While their training data includes extensive high-level evidence from clinical literature, a substantial portion is derived from unlabeled internet-sourced text, making them susceptible to perpetuating and amplifying inherent biases present in the data [8,10,11,14,21,22]. A more critical concern in clinical settings is the propensity of LLMs to generate responses even in the absence of sufficient information, a phenomenon known as hallucination. Such hallucinations can lead to deviations from established medical practices, posing danger for clinical decision-making [11,13,14,22].

Following this reasoning, Retrieval-Augmented Generation (RAG) has emerged as a promising solution for dealing with these shortcomings, enhancing the accuracy of LLMs and significantly reducing hallucinations, while offering increased flexibility and adaptability to new knowledge. Unlike fine-tuning, which requires costly, time-intensive retraining whenever new data becomes available, RAG integrates external and reliable sources at query time, allowing models to remain updated without repeated re-optimization [23,24,25,26,27,28,29]. This makes RAG a more efficient and scalable alternative for dynamic domains such as clinical decision-making. A key strength of RAG lies in its ability to retrieve specialized knowledge tailored to the clinical context [24,26,30,31,32,33,34]. The efficacy of RAG has been demonstrated in studies such as that by Lammert et al., whose MEREDITH RAG model provided appropriate treatment recommendations based on specific biomarkers, achieving 0% disagreement among oncologists [35].

Despite these promising features, the effectiveness of RAG is not absolute. Its performance is highly dependent on the quality and structure of the information retrieved, and limitations such as fragmented context, irrelevant passages, and noisy outputs can still compromise accuracy [24]. If the segmentation (or “chunking”) breaks procedural steps or safety qualifiers across boundaries, the retriever may surface partial or misleading passages, ultimately compromising the reliability of the model’s response. These retrieval issues underscore the need to carefully optimize RAG’s architectural components, especially in high-stakes applications like clinical decision support.

One of the most critical levers for addressing these limitations is chunking, which is the process of dividing information into smaller, retrievable units that can be stored and accessed by RAG systems [36]. Chunking determines whether responses are contextually coherent or fragmented by directly shaping what RAG retrieves. Naive chunking strategies, which split text into arbitrary equal-sized segments, often dilute meaning and surface incomplete or misleading information. This reduces retrieval precision and undermines the safety and reliability of clinical applications, where overlooking a key detail can have serious consequences [26,37,38,39,40]. Advanced chunking methods, such as semantic, proposition-based, and adaptive approaches, offer a path forward by organizing text around meaningful units rather than token counts, preserving context and improving retrieval accuracy. In this way, chunking directly complements RAG’s strengths, helping to overcome its retrieval bottlenecks and ensuring that the information delivered is relevant and trustworthy.

Semantic chunking divides text by meaning rather than length, while methods like SimilarSentenceSplitter cluster related sentences to preserve coherence [36,41]. Proposition chunking goes further by breaking down content into atomic fact-based units, each self-contained and precise, which research shows significantly improves retrieval accuracy [36,42]. Adaptive chunking adds flexibility by tailoring chunk size to the content’s structure, maintaining logical boundaries such as sentences or sections [43,44]. Together, these methods enhance the performance of RAG systems by minimizing hallucinations, improving context preservation, and delivering accurate, contextually relevant information, which is particularly vital in high-stakes fields like medicine.

While several studies have explored retrieval optimization or prompt engineering in RAG systems, few have isolated chunking as an independent determinant of retrieval precision or contextual fidelity [26,37,38,39,40]. Most prior work either uses fixed-length segmentation without justification or introduces semantic or proposition-based methods without quantifying their clinical relevance [36,41,42]. Consequently, there remains limited evidence on how chunk boundary policy alone influences the accuracy, completeness, and safety of responses in real clinical scenarios. This gap is particularly relevant for decision-support contexts, where incomplete retrieval can translate into misleading guidance.

The present study addresses these shortcomings through the following contributions:

- Isolated evaluation of chunking strategies: Four otherwise identical RAG pipelines were built that differed only in how documents were segmented—allowing direct measurement of chunking effects independent of model or retriever bias.
- Definition of an adaptive chunking method: A flexible policy combining similarity thresholds and variable window sizes was implemented to preserve topic continuity while avoiding information dilution.
- Quantitative benchmarking using real clinical data: Thirty representative postoperative rhinoplasty queries were used to evaluate accuracy, relevance, and information-retrieval metrics under controlled conditions.
- Model-agnostic optimization: Improvements were achieved without retraining or fine-tuning the language model, demonstrating an off-model route to performance gains.
- Transparency and reproducibility: Full parameter settings, evaluation scales, and statistical procedures are disclosed to facilitate replication and future comparisons.

Collectively, these elements establish a clear link between document segmentation policy and safe, contextually reliable performance in clinical RAG systems.

This paper tests and compares these three advanced chunking techniques against a basic token-fixed approach for clinical decision support for postsurgical support after rhinoplasty procedures. We hypothesize that the advanced chunking techniques, especially the Adaptive chunking, will outperform the baseline in all tested metrics. We benchmarked these approaches’ performance on 30 common patient complaints post-rhinoplasty from our institution.

## 2. Methods

### 2.1. Study Design and Setting

A controlled experimental design was employed to compare four different document chunking strategies within a RAG pipeline for clinical decision support. A single authoritative rhinoplasty reference document was created merging “Essentials of Septorhinoplasty: Philosophy, Approaches, Techniques,” Postoperative Care and Management; “Plastic Surgery: A Practical Guide to Operative Care,” Rhinoplasty; “Rhinoplasty Cases and Techniques,” Postoperative Care; “Plastic Surgery, Volume 2: Aesthetic Surgery (Fifth Edition),” Open Rhinoplasty Technique, Closed Rhinoplasty Technique, and Secondary Rhinoplasty. This document served as the knowledge corpus.

System performance was evaluated on a set of 30 representative post-rhinoplasty clinical queries, ranging from routine postoperative care questions to potential complication scenarios, compiled by domain experts based on common concerns encountered at our institution. Each query was processed using the same RAG system configuration (Gemini-1.0 Pro, an in-house large language model integrated with the rhinoplasty knowledge base), and the only difference between experimental conditions was the chunking method used to preprocess the knowledge document. For each query, the system retrieved relevant text chunks from the pre-chunked document via vector similarity search, then fed those chunks into the large language model to generate an answer. Two independent medical doctors evaluated the returned answers and their retrieved evidence for relevance and accuracy against the reference document.

### 2.2. Document Chunking Strategies

**Figure 1:**
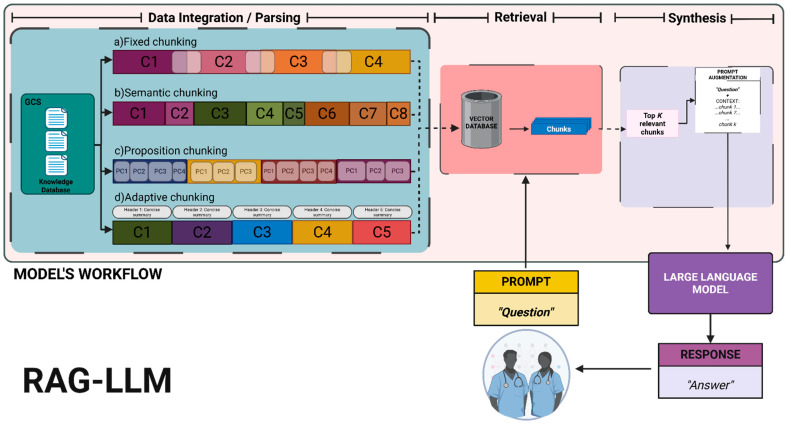
Document chunking strategies evaluated and entire pipeline representation. (1) Recursive character-based (Fixed-size) splits contents at arbitrary token counts, risking cross-section cuts. A paragraph → sentence → punctuation hierarchy was adopted with ≈1000-character maximum and ≈100-character overlap. (2) Semantic cluster chunking forms coherent, topic-based units, splitting at meaning shifts, where sentences were embedded with TF-IDF and grouped via k-means (*k* = 6); each cluster is concatenated in original order. (3) Proposition-based chunking indexed atomic, claim-level statements for high-precision retrieval. The approach leveraged an LLM that extracts atomic propositions per sentence (temperature ≈ 0.2; max ≈ 256 tokens), which are grouped into chunks until ≈500-word capacity or topic shift. (4) Adaptive chunking aligns to section and sentence boundaries with variable window sizes and backtracking to avoid mid-sentence cuts. Sentence embeddings (all-MiniLM-L6-v2) with cosine similarity > 0.8 extend the current chunk; a ≈ 500-word cap starts a new chunk; concise micro-headers (BART summarizer) are prepended to each finalized chunk. C#: Chunk number; each type of chunking is independent. Created in Biorender. Cesar A. Gomez-Cabello (2025) https://BioRender.com/062tua0 [45].

Four document chunking strategies were explored for segmenting the rhinoplasty reference document before indexing. In all cases, the goal was to create text chunks that were coherent and of a suitable size for retrieval while preserving as much contextual meaning as possible. The following sections detail the four document chunking strategies evaluated in this study, and Figure 1 provides a graphic summary.

#### 2.2.1. Recursive Character-Based Chunking

The document was first segmented using a recursive character splitter that respects natural text boundaries. In practice, the text was split by paragraphs; if a paragraph exceeded approximately 1000 characters, it was further broken down into smaller units at sentence boundaries. If a sentence was still too long, splits at subordinate punctuation (commas, semicolons, etc.) were applied recursively until all chunks were ≤1000 characters long.

A maximum chunk size of roughly 1000 characters was chosen to ensure each chunk remained within the model’s input limits while capturing a complete thought or section of text. A fixed overlap of about 100 characters was implemented between consecutive chunks to maintain context continuity between chunks. This means that the ending portion of one chunk is repeated at the beginning of the next chunk, which helps prevent any important sentence or clause from being lost at the boundary. This overlap strategy mirrors the behavior of the RecursiveCharacterTextSplitter (as in LangChain) with a chunk_size of approximately 1000 and a chunk_overlap of about 100, providing continuity for the model when processing sequential chunks [46].

#### 2.2.2. Semantic Cluster Chunking

The second strategy segmented the document into semantically coherent sections by leveraging clustering. The document was first split into individual sentences using spaCy, and each sentence was then represented as a TF-IDF feature vector (using scikit-learn’s TfidfVectorizer with default settings, i.e., no custom stop-word list). Next, K-means clustering was applied to group these sentence vectors into *k* clusters based on content similarity. A value of *k* = 6 was chosen for clustering, aiming to produce about six thematic chunks from the document. This choice was guided by the intuition that the reference document could be broadly divided into a handful of major topical sections; using six clusters ensured each cluster would be topically coherent without becoming too broad or too granular. (If the document had fewer than six sentences, *k* was equal to the sentence count to avoid empty clusters.) The random seed (random_state = 42) was fixed for the K-means algorithm to ensure the clustering result was reproducible. All sentences assigned to the same cluster were then concatenated in their original order to form one chunk per cluster.

This method produces chunks containing topically related information, preserving thematic coherence within each chunk [47]. By clustering at the sentence level using TF—IDF, this approach ensured that each resulting chunk centered on a distinct subtopic of the rhinoplasty reference material.

#### 2.2.3. Proposition-Based Chunking

The third strategy employed a large language model to segment text by logical propositions rather than by surface features like character count or raw semantic similarity. The process began by splitting the document into sentences with spaCy. Then, for each sentence, an LLM such as Vertex AI text-bison@002 was used to extract the distinct propositions or factual statements contained in that sentence. In other words, the model was prompted to break down complex sentences into bullet-style atomic assertions, with each proposition representing a single complete idea or claim. The LLM’s generation parameters were configured to favor consistency and completeness: a relatively low temperature of 0.2 to minimize randomness and a maximum of 256 tokens for the output of each sentence to ensure the model could list all propositions in a long sentence if needed. This step resulted in a sequence of concise proposition statements capturing the fine-grained meaning units of the text.

These propositions were then grouped into coherent chunks. From the first proposition, subsequent propositions were appended to the current chunk until adding one more would make the chunk exceed roughly 500 words in length (a limit chosen to keep chunks sufficiently small for the model’s context window). Once a chunk reached that size limit, or if a new proposition was unrelated to the current chunk’s focus, a new chunk was started. This process yielded highly granular, meaning-focused segments, since each chunk contains one or a few closely related propositions from the text. By leveraging the LLM to identify logical breakpoints, this method aligned the chunks with the document’s inherent assertions and ideas, resulting in fine-grained segments that remain meaningful on their own [48].

#### 2.2.4. Adaptive Chunking

The fourth method was a novel adaptive chunking approach developed in this study, which dynamically adjusted chunk boundaries based on semantic similarity and content structure, and annotated chunks with brief headers for context. The document was tokenized into sentences using spaCy, and a semantic embedding for each sentence was computed using a SentenceTransformer model (all-MiniLM-L6-v2). These embeddings allowed measurement of the semantic relatedness between sentences. Chunks were then built iteratively in a single pass through the document. A new chunk was initiated with the first sentence, and for each subsequent sentence, a decision was made whether to continue the current chunk or start a new chunk based on two criteria:

(i) Semantic continuity: The cosine similarity was calculated between the embedding of the new sentence and that of the last sentence in the current chunk or an aggregate embedding of the current chunk. When the similarity score exceeded 0.8 in this implementation, the incoming sentence was treated as a continuation of the current topic and appended to the active chunk.
(ii) Chunk length: It was checked whether adding the new sentence would keep the chunk under a maximum length of about 500 words. The chunk would be broken if the semantic similarity fell below 0.8 or the length limit was exceeded by adding that sentence. The current chunk was finalized, and a new chunk was started with the new sentence. This adaptive approach produced chunks of varying size that each maintained high internal coherence; sections of text that stayed on a consistent topic naturally formed longer chunks up to the length cap, whereas a rapid topic change triggered a smaller chunk, aligning chunk boundaries with shifts in content [49].

An additional feature of this adaptive method was the use of dynamic micro-headers to provide context for each chunk. Whenever a chunk was finalized (especially if it ended in the middle of a section or thematic unit), a short descriptive heading was automatically generated for that chunk before proceeding. Specifically, a BART-based summarization model, such as facebook/bart-large-cnn, was prompted to produce a one-line summary or title for the chunk’s content. The summarizer was run in deterministic mode (no random sampling) to ensure consistency, and the output length was constrained to be very concise (roughly 5–15 words) so that the header captured the gist of the chunk. For example, a chunk detailed in postoperative care might receive a header like Post-Operative Care Instructions. These AI-generated headers were prepended to the chunk text and treated as part of the chunk’s content for retrieval purposes to make each chunk self-explanatory in isolation. This technique resembles an agentic chunker approach that dynamically annotates segments with metadata for additional context. In rare instances when the summarization model could not produce a header due to a processing error or because the chunk was too brief, a default placeholder reading “no header available” was inserted to ensure that each chunk retained a title. By adaptively adjusting chunk boundaries and adding informative headers, this method aimed to maximize each chunk’s coherence and usefulness for retrieval [50].

##### Improvement Steps and Limitations

The adaptive chunking process was developed iteratively from the semantic and proposition frameworks described earlier. Each pilot iteration adjusted one factor—boundary policy, similarity threshold, or summarization method—while keeping the RAG stack constant. Early versions without micro-headers improved recall but reduced precision due to topic drift; adding concise summaries corrected this by tightening query alignment. Lower similarity thresholds (<0.7) caused excessive merging of unrelated content, while stricter ones (>0.85) oversplit instruction units. The final configuration (cosine similarity ≥0.8, 500-word cap, and micro-headers) balanced completeness and specificity. Despite these refinements, adaptive chunking remains sensitive to corpus density and sentence embedding quality; rapid topic shifts or sparse transitions can still yield uneven chunk lengths and rare boundary errors. In future work, we will evaluate adaptive policies that adjust similarity thresholds dynamically based on local semantic variance or discourse markers to further stabilize chunk boundaries.

#### 2.2.5. Parameter Justification

During pilot testing, the recursive character approach with a 1000-character window and 100-character overlap offered the most balanced outcome. Larger window sizes tended to introduce redundancy, which reduced precision, while smaller windows disrupted multi-sentence instructions, lowering recall. In semantic clustering, a target of six clusters was selected to align with the document’s major thematic divisions. Using more clusters fragmented key caveats and weakened completeness, whereas fewer clusters merged unrelated topics, diluting focus. For proposition-based segmentation, setting the temperature at 0.2 and limiting output to 256 tokens helped maintain concise, verifiable statements without paraphrastic drift, while a 500-word limit on chunk size reduced the reconstruction burden during answer generation. Finally, in the adaptive method, a similarity threshold of 0.8 prevented topic bleeding without oversplitting, and a 500-word cap ensured that directive, timing, and exception elements were preserved together, keeping postoperative instructions intact and clinically meaningful. These values were derived from pilot runs that iteratively varied threshold and window size while holding all other parameters constant. Lower thresholds (<0.75) allowed topic bleed across unrelated guidance, decreasing precision, whereas higher thresholds (>0.85) fragmented multi-sentence instructions and reduced recall. Similarly, increasing the word cap beyond 500 introduced redundancy and repetition, while smaller caps (<300 words) disrupted directive, timing, and safety qualifiers that must remain within a single retrievable span.

After applying the above chunking strategies, the resulting chunks were indexed for use in the RAG system. Importantly, aside from the chunking step, the rest of the pipeline was kept constant across all conditions to enable a fair comparison.

In every case, each chunk was converted into a 768-dimensional embedding using the Vertex AI text-embedding-004 model. This embedding model was chosen for its strong performance on semantic text representations, and its 768-dimensional vectors provided a high-resolution semantic encoding of the chunk content. All chunk embeddings across all strategies were stored and indexed in a Chroma vector database optimized for efficient cosine similarity search.

At query time, the system performed a similarity search in this vector index to retrieve the top-*k* most relevant chunks for the given question, with *k* = 5 in this implementation. The choice of five chunks provided a balance between supplying the language model with enough relevant context covering different aspects of the answer and avoiding introducing too much irrelevant or redundant text. The retrieved chunks were then fed into the Gemini-1.0 Pro LLM as augmented context, and the LLM generated a final answer to the clinical query based on both the question and the retrieved knowledge.

This unified retrieval-and-generation pipeline was identical for all chunking methods, ensuring that any differences in answer quality could be attributed to the chunking strategy alone. By evaluating the responses and their supporting evidence for relevance and accuracy, it was possible to assess how each chunking approach affected the RAG system’s ability to find and present correct information for post-operative decision support. The controlled setup and consistent pipeline across conditions provided a robust basis for comparing the four chunking strategies regarding downstream retrieval effectiveness and answer quality.

#### 2.2.6. Pipeline Configuration and Model Parameters

The complete Retrieval-Augmented Generation (RAG) configuration is summarized below to ensure transparency and reproducibility.

- Backbone LLM: Gemini 1.0 Pro (no fine-tuning).
- Embeddings: Vertex AI text-embedding-004 (768-D), vectors L2-normalized; cosine similarity used for nearest-neighbor retrieval.
- Vector Store/Retrieval: Chroma database with an HNSW (hnswlib) approximate-nearest-neighbor index for dense retrieval.
- Lexical Baseline: Standard BM25 ranker (*k*_1_ = 1.2, b = 0.75) reported separately; not fused with dense retrieval.
- Chunk Source (Adaptive): Cosine-similarity threshold = 0.80; target span ≈ 500 words; minimum ≈ 60 words; optional overlap ≤ 30 tokens during boundary repair; continuity checked against both last-sentence and span-centroid embeddings; linker-aware repair for/until/unless/after/within; micro-headers generated heuristically from noun-phrase titles.
- Fixed-window Baseline: 250/400/600 spaCy-token spans with 50-token overlap; the best-performing fixed size is shown in the main figures, with the full sweep reported in the Supplement.
- Prompting (single-stage): A fixed system prompt instructed the model to preserve instruction-unit fidelity (step, timing, safety), include citation markers when available, and defer when uncertain. No self-querying or multi-stage prompt optimization was used.
- Generation Limits: Context window ≈ 6000 tokens and output cap ≈ 600 tokens. Decoding used deterministic (temperature = 0) or low-variance sampling to improve stability and reduce hallucinations.
- Evaluation: Metrics included precision, recall, F_1_, and non-response rate; blinded clinical ratings assessed accuracy, completeness, readability, and actionability. Mean values are reported with 95% bootstrap confidence intervals.
- Rationale for Gemini Settings: A single-stage prompt and conservative decoding were chosen to align responses tightly with clinical instructions and minimize variability. The 6 k-token context and 600-token output cap follow Gemini documentation and pilot stability checks: larger contexts or longer generations increased verbosity without improving grounded accuracy or blinded ratings. These values were fixed across all runs to ensure comparability.

### 2.3. Evaluation Tools and Outcome Metrics

The quality of the generated answers and the retrieval system’s performance were evaluated to assess the effectiveness of the four chunking strategies. Two medical experts independently rated each response’s medical accuracy and relevance using three-point scales. Ratings were reviewed jointly after the initial pass, and any differences were discussed until a consensus was reached. Formal inter-rater reliability (e.g., κ or ICC) was not computed, as the aim of this phase was to establish proof of concept rather than to quantify agreement; a structured consensus review was therefore used to ensure rating consistency. A 3-point Likert scale was selected to preserve clarity in expert evaluation. Because the outcomes represented categorical judgments of factual correctness and relevance rather than subjective intensity, a compact three-level scale was deemed conceptually appropriate. Broader categorical schemes are commonly used in clinical language-model evaluation studies where the goal is to determine whether a response is correct, partially correct, or incorrect, rather than to capture subtle preference gradations. This approach emphasizes interpretability and avoids false precision when assessing factual accuracy. For medical accuracy, a score of 1 indicated content that conflicted with accepted clinical practice; a score of 2 reflected responses that combined correct and incorrect elements; and a score of 3 denoted guidance consistent with standard references and routine care. Similarly, for relevance, 1 signified that the response content was irrelevant to the question, 2 captured broadly related answers that lacked specific direction, and 3 indicated content that directly addressed the clinical scenario with actionable recommendations.

Ground-truth information for evaluating the answers was drawn from the same authoritative rhinoplasty resources used to build the knowledge database, including key chapters on postoperative management in Essentials of Septorhinoplasty, Plastic Surgery: A Practical Guide to Operative Care, Rhinoplasty Cases and Techniques, and Plastic Surgery, Volume 2: Aesthetic Surgery (Fifth Edition).

Each chunking strategy’s retrieval performance was further evaluated using standard information retrieval measures: precision, recall, and F1. Precision (TP/(TP + FP)) is the proportion of retrieved chunks that contain authoritative, relevant information. Recall (TP/(TP + FN)) is the proportion of all relevant passages successfully retrieved. F1 (2 × precision × recall/(precision + recall)) is the harmonic mean of precision and recall, providing a balanced performance measure. A chunk was counted as a true positive if it matched the authoritative references on the topic, a false positive if it did not correspond to the authoritative sources or was irrelevant to the scenario, and a false negative if a relevant chunk was not retrieved. Thus, higher precision indicates fewer off-topic chunks, higher recall indicates better coverage of pertinent content, and a higher F1 denotes a more balanced retrieval system.

### 2.4. Statistical Analysis

Descriptive statistics (mean and standard deviation, SD) for the accuracy and relevance ratings were calculated using Microsoft Excel (Version 2503, Build 16.0.18623.20266, 64-bit). To compare performance across the four chunking methods, a one-way ANOVA was performed, and Tukey post hoc tests were conducted for pairwise differences where applicable, with significance defined as *p* < 0.05. Formal tests of normality and homogeneity of variances were not performed. Given the balanced design (30 items per group) and the known robustness of one-way ANOVA to moderate assumption violations, we complemented *p*-values with bootstrap 95% Cis and effect sizes to contextualize uncertainty and magnitude. To assess sampling variability, 95% confidence intervals (CI) were calculated using nonparametric bootstrap resampling (1000 iteration, sampling with replacement) for each chunking strategy and outcome. Beyond *p*-values, effect sizes were reported to quantify the magnitude of differences: eta-squared (η^2^) for the ANOVA main effect) and Cohen’s d for pairwise contrasts using pooled standard deviations). Effect sizes were interpreted with conventional thresholds (η^2^: small ≈ 0.01, medium ≈ 0.06, large ≈ 0.14; d: small ≈ 0.2, medium ≈ 0.5, large ≈ 0.8). For information-retrieval metrics (precision, recall, F1), inferential tests were not applied because these metrics are deterministic over a fixed benchmark and illustrate practical retrieval differences rather than hypothesis-driven comparisons; instead, uncertainty was expressed via nonparametric bootstrap CIs (1000 iterations, resampling the 30 items), and results are reported as means with 95% CIs.

## 3. Results

### 3.1. Medical Accuracy

**Figure 2:**
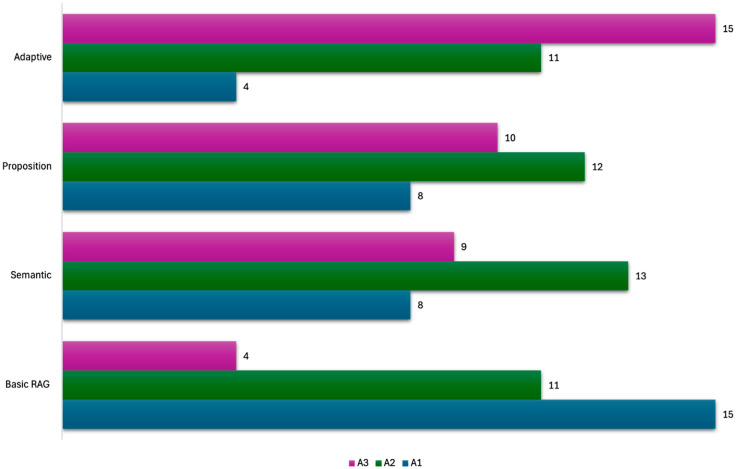
Bar graph showing the models’ accuracy score on a 3-point Likert scale. A3: Likert score of 3 points; A2: Likert score of 2 points; A1: Likert score of 1 point.

The model implementing adaptive chunking achieved the highest mean medical accuracy score (2.37 ± 0.72) on the three-point Likert scale, with a 95% bootstrap CI of 2.10–2.60. Of the 30 clinical questions evaluated, 50% (n = 15) of responses were rated completely accurate, while 87% (n = 26) were at least somewhat accurate. This performance was significantly superior to the basic model implementing a fixed-size chunking strategy (*p* = 0.001). The proposition-based model obtained a mean score of 2.07 ± 0.78 (95% CI 1.80–2.33), with 33% (n = 10) of responses fully accurate and 73% (n = 22) at least somewhat accurate. In contrast, the semantic model reached 2.03 ± 0.76 (95% CI 1.77–2.33), with 30% (n = 9) fully accurate and 73% (n = 22) at least somewhat accurate. Differences between the proposition-based or semantic models and the basic model did not reach statistical significance. Conversely, the basic model’s mean accuracy was 1.63 ± 0.72 (95% CI 1.40–1.90), with only 13% (n = 4) of responses fully accurate and 50% (n = 15) at least somewhat accurate. The overall ANOVA showed a moderate effect size (η^2^ = 0.11). Pairwise comparisons indicated a large improvement for adaptive versus basic (d = 1.03) and moderate improvements relative to semantic (d = 0.46) and proposition-based (d = 0.40) models. Figure 2 shows the comparison of the models’ accuracy and Table 1 the confidence intervals.

Increasing the proportion of completely accurate answers from 13% (50%) with basic fixed-size chunking to 50% (87%) with adaptive chunking underscores a meaningful improvement in the safety and reliability of model-generated guidance for clinical decision support.

### 3.2. Clinical Relevance

**Figure 3:**
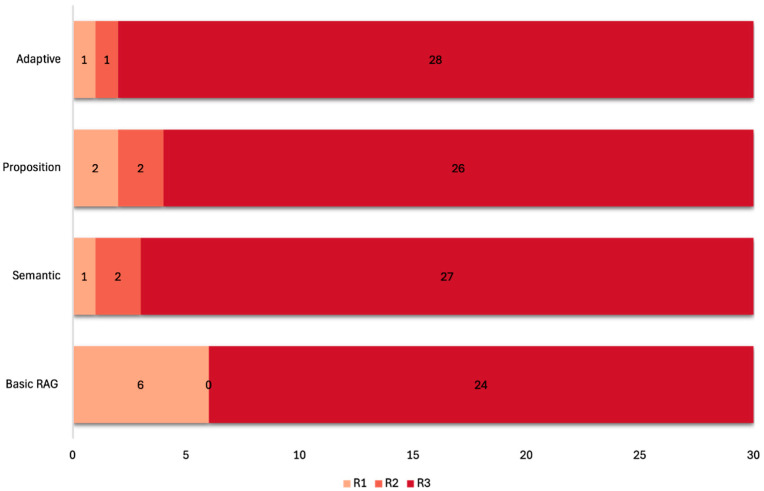
Stacked bar chart showcasing the models’ relevance scores on a 3-point Likert scale. R1: relevance score of 1; R2: relevance score of 2; R3: relevance score of 3.

As with medical accuracy, the adaptive chunking model achieved the highest clinical relevance, with a mean Likert score of 2.90 ± 0.40 (95% bootstrap CI 2.73–3.00). Of the 30 questions, 93% (n = 28) of responses were fully relevant, and 97% (n = 29) were at least somewhat relevant. The semantic model closely followed with a mean of 2.87 ± 0.43 (95% CI 2.70–3.00), yielding 90% (n = 27) fully relevant responses and 97% (n = 29) at least somewhat relevant. The proposition-based model achieved 2.80 ± 0.55 (95% CI 2.57–2.97), with 87% (n = 26) fully relevant and 93% (n = 28) at least somewhat relevant. Conversely, the basic fixed-size chunking model had a mean of 2.60 ± 0.81 (95% CI 2.33–2.87), with 80% (n = 24) fully relevant and 80% (n = 24) at least somewhat relevant. Pairwise effect sizes (Cohen’s d) reflected a moderate advantage for adaptive versus basic (d = 0.45) and small to negligible differences relative to semantic (d = 0.07) and proposition-based (d = 0.20) models, aligning with the overlapping bootstrap Cis (Table 1). Figure 3 illustrates the relevance of the models’ responses.

Although there was no statistically significant difference between the different chunking strategies, beyond higher point estimates, variability decreased with advanced chunking (i.e., SD narrowing from 0.81 with basic to 0.40–0.55), suggesting greater stability and a higher likelihood of surfacing directly actionable content. By increasing the proportion of fully relevant responses from 80% to 93% and tightening variability, adaptive chunking can reduce review burden and support faster, more consistent clinical decisions.

### 3.3. Information-Retrieval Performance

**Figure 4:**
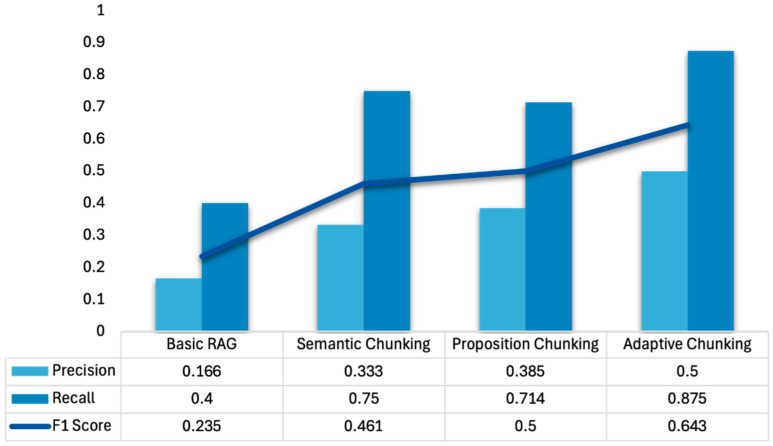
Information retrieval of the models. Dark blue bars showcase recall, light blue bars demonstrate precision, and the navy blue line shows F1 score (harmonic mean of precision and recall). Metrics are descriptive, and definitions are detailed in the Methods section.

As with medical accuracy and clinical relevance, the adaptive chunking model achieved the highest information-retrieval performance, with a precision of 0.50 (95% CI 0.31–0.68), a recall of 0.88 (0.69–1.00), and an F1 score of 0.64 (0.36–0.78). In practical terms, one in two retrieved passages was relevant, and nearly nine in ten relevant items were captured. The proposition-based model followed with 0.39 (0.21–0.57), recall 0.71 (0.46–0.93), and an F1 of 0.50 (0.25–0.67), while the semantic model reached 0.33 (0.16–0.52), recall 0.75 (0.50–1.00), and F1 0.46 (0.19–0.64). Conversely, the basic fixed-size chunking model performed lowest, with 0.17 (0.04–0.32) precision, 0.40 (0.10–0.70) recall, and an F1 of 0.24 (0.00–0.39), indicating that fewer than one in five retrieved passages were relevant and less than half of the relevant evidence was found. Relative to baseline, adaptive chunking improved precision by +0.33, recall by +0.48, and F1 by +0.42. Figure 4 provides a graphical comparison of these results, while Table 1 compares the models’ CIs.

These findings indicate that advanced chunking, especially adaptive chunking, broadens coverage of clinically relevant evidence (higher recall) while reducing noise (higher precision). In practice, these gains lower the reviewer burden and decrease the risk of missing key guidance, providing faster, safer, and more consistent clinical decision support.

Evaluating each segmentation method in isolation reveals that proposition- and semantic-based chunking each contributed distinct advantages but also clear constraints. Proposition-level segmentation improved precision by tightly aligning retrieval units with discrete statements but fragmented context, occasionally omitting modifiers essential to safety or timing. Semantic grouping improved recall by preserving topic continuity but risked redundancy when similar sentences clustered across chunks. Adaptive chunking integrated both principles—semantic continuity with proposition-level closure—through dynamic boundary adjustment and micro-header reinforcement, which likely explains its superior balance of precision and recall. The stepwise improvements observed across models suggest that progressively aligning chunk boundaries with clinical discourse elements yields more complete and contextually stable retrieval without the noise accumulation typical of fixed-length windows.

Across accuracy, relevance, and retrieval, the adaptive approach increased the proportion of fully correct and fully relevant answers, reduced variability, and improved both precision and recall. In practice, these improvements translate into fewer non-actionable passages for clinicians to review, directly lowering cognitive load and shortening the time needed to reach a safe, evidence-grounded recommendation. The greater recall achieved by adaptive chunking—capturing nearly nine of ten relevant items—reduces the likelihood of missing safety qualifiers or escalation triggers, while its higher precision ensures that retrieved content is more clinically useful. Together, these gains support faster and more consistent clinical decision-making, minimizing review burden and the risk of complication oversight.

To illustrate common failure modes across segmentation strategies, several representative examples are provided in Supplementary Table S1. These examples show how fixed windows truncated timing and safety qualifiers, semantic clustering separated exception clauses, and proposition-based segmentation fragmented instruction units. Adaptive chunking preserved full directive–timing–safety relationships within single spans, producing more coherent and contextually complete responses that aligned with observed gains in accuracy and F_1_.

## 4. Discussion

### 4.1. Interpretation and Significance

In retrieval-augmented generation for clinical decision support, segmentation is not a cosmetic pre-processing step; boundary placement determines what evidence is retrieved, how it is contextualized, and whether it is ultimately used to guide care [26,51,52]. We evaluated this premise directly by holding the retrieval stack constant—knowledge base, embedding model, language model and top-k—while varying only the chunking method across four strategies in a controlled comparison of thirty real postoperative rhinoplasty queries. This led to distinct performance patterns. Adaptive, embedding-guided chunking with dynamic micro-headers provided the most reliable guidance, achieving the highest information-retrieval scores (precision 0.50, recall 0.88, F1 = 0.64) and the best medical accuracy (mean 2.37 ± 0.72; 50% fully accurate; 87% at least somewhat accurate) and clinical relevance (mean 2.90 ± 0.40; 93% fully relevant; 97% at least somewhat relevant) outcomes. Proposition-level segmentation retrieved granular, discriminative facts and performed well on narrow questions, but sometimes lost the framing required for multi-part queries. Semantic clustering with TF-IDF and K-means improved topical focus and raised precision and recall over the baseline, yet it occasionally broke narrative links that should remain together for appropriate clinical judgement. Fixed recursive character windowing performed worst, fragmenting coupled statements, duplicating content across overlapping windows, and yielding the lowest precision (0.17), recall (0.40), and F1 (0.24). The only statistically significant difference in accuracy was observed between adaptive and basic fixed-size chunking (*p* = 0.001); other pairwise comparisons did not reach significance within this design.

These results are consistent with the way postoperative guidance is authored and used. In the source corpus, surgeons frequently encode instruction, timing, and exception clauses as a compact unit, and retrieval succeeds when that unit is preserved intact. Methods that split these elements across boundaries depress recall or force the retriever to assemble multiple pieces within a fixed top-k, a pattern consistent with classic findings that segmentation quality affects downstream passage retrieval [53,54]. Overlapping windows compound the problem by producing near-duplicates that inflate false positives and consume limited retrieval slots with redundant hits [55,56]. The net effect for fixed windows is diluted embeddings that are similar to many queries yet not specific enough to fully answer them, reflected in low precision, low recall, and the weakest F1. In contrast, strategies designed to preserve contextual integrity and thematic coherence produced stepwise improvements across metrics, culminating in the adaptive method, which maintained both semantic cohesion and completeness within each evidence unit [46,57,58].

Semantic clustering and proposition-level segmentation illustrate complementary failure modes. Clustering raises topical purity by grouping sentences that share a theme, which increases the chance that a chunk is “about the right thing,” thereby improving precision to roughly 0.33 and recall to approximately 0.75 [59,60]. However, by optimizing for intra-cluster coherence, the method can splice apart cross-sentence dependencies, especially if/when caveats and escalation thresholds that reside just outside the cluster boundary, yielding answers that are relevant but incomplete [61,62,63]. Proposition-level segmentation pushes in the opposite direction by indexing atomic assertions at the sentence or sub-sentence level [48,64,65]. This granularity increases discriminability and benefits single-fact questions, with precision rising toward 0.38, but it burdens retrieval for composite questions [66]. Under a fixed top-k budget, micro-facts scatter, and the generator must reconstruct the relationships and conditional logic among them (“additional reference”); reviewers described resulting answers as accurate in isolated points yet lacking the connective narrative needed to prioritize and contextualize advice [58].

Adaptive chunking balanced these trade-offs. It grew each chunk sentence by sentence while local semantics remained stable (cosine similarity ≥ 0.8) and stopped at a modest length budget (~500 words), producing segments that were tightly focused and self-contained [67]. Because each segment tended to include the directive, its timing, and any exceptions, a single retrieved span often satisfied the query without post hoc assembly. The integration of concise micro-headers further narrowed the phrasing gap between patient questions and guideline terminology, increased matchability without inflating token counts, and improved interpretability during expert review [49,50,68]. The combined effect was a simultaneous increase in recall, by keeping instruction units intact, and in precision, by avoiding topic dilution and near-duplicate inflation. This ultimately led to the observed gains in F1, graded accuracy, and relevance.

In discussing the comparative performance of the different chunking strategies, a clear pattern emerged. Adaptive segmentation consistently provided the strongest results because it preserved directive, timing, and exception information together within a single unit, allowing most queries to be answered with one coherent span rather than requiring the model to piece together fragments from multiple chunks. Semantic clustering improved topical alignment by grouping related content, but its tendency to separate cross-topic qualifiers, such as important caveats or conditional clauses, meant that some safety-critical context was occasionally lost. Proposition-level indexing, while valuable for generating highly granular and auditable fact units, placed the burden of reconstructing full clinical instructions onto the generator, particularly under a limited top-k retrieval budget. This trade-off often produced answers that were factually correct but fragmented in narrative flow. Together, these findings highlight why adaptive chunking outperformed the alternatives, as it offered the best balance between semantic focus, contextual completeness, and clinical reliability.

### 4.2. Comparison with the Existing Literature

Two recurrent themes in the literature align with our findings. First, retrieval quality—not prompt style alone—emerges as the dominant determinant of factuality, safety, and usability in RAG settings. When retrieval is poorly aligned with the clinical need, even strong language models drift; contrastingly, when retrieval is precise and complete, generated answers become more faithful, verifiable, and efficient to review [46,58]. Similarly to the findings by Lewis et al., who showed that coupling generation to retrieved evidence improves faithfulness on knowledge-intensive tasks, and Zakka et al., tethering clinical responses to authoritative sources to improve verifiability, our head-to-head study isolates the retrieval channel as causal for quality: changing only the boundary policy (with the corpus, embeddings, LLM, and k held constant) yielded large, consistent differences in accuracy, relevance, and F1 [26,52]. In line with Demner-Fushman and Lin, who demonstrated gains in clinical question answering when evidence selection is optimized, our results indicate that better-formed retrieval units act as a force multiplier on downstream answer quality [69]. Comparable to Wang et al., who observed that consistent passage segmentation followed by reranking improves open-domain QA, we similarly observed stepwise improvements when moving from fixed windows to more coherent chunking, with the most significant gains under the adaptive policy [60].

Second, topical coherence and boundary integrity are preconditions for effective retrieval. Consistent with Hearst, who introduced topic-based text segmentation and showed that misplaced boundaries degrade cohesion cues, our data show that arbitrary fixed windows dilute embeddings and inflate false positives, especially when overlapping windows duplicate content, and thereby depress precision and recall relative to more coherent units [53,56]. Our findings differed from those of Tiedemann, who evaluated alternative segmentation strategies primarily from an IR efficiency standpoint; in our clinical setting, strategies that optimize intra-chunk purity alone (e.g., simple clustering) can harm completeness by splitting qualifiers and escalation thresholds across boundaries [62]. Similarly to Rindflesch and Fiszman and to Stanovsky and Dagan, we note that proposition- or relation-level indexing improves retrieval of single facts; however, our results extend that line of work by showing that composite, multi-clause postoperative questions suffer under fixed top-k budgets because micro-facts scatter and must be recomposed in a long context, a setting already known to be position-sensitive per Liu et al. [64,65,66]. Finally, akin to Nogueira et al.’s document-expansion approach, our micro-headers narrow phrasing gaps between queries and source text; unlike expansion that predicts additional queries for whole documents, our headers are span-scoped and lightweight, improving matchability without inflating tokens [68].

Taken together, these comparisons suggest that our contribution is orthogonal to prompt engineering and complementary to prior retrieval optimizations: by aligning chunk boundaries with the minimal clinical unit of meaning (directive, timing, and exception in one span), adaptive chunking preserves completeness and coherence, reproducing the benefits reported by prior work on evidence-tethered generation [26,52] while avoiding the boundary-induced failure modes documented in topic segmentation and long-context studies [53,66]. The addition of micro-headers resonates with evidence that phrasing gaps between queries and documents degrade match quality, with brief, domain-specific descriptors reducing that gap without requiring elaborate prompt engineering [49,50].

### 4.3. Importance and Implications for Medical Practice

Clinical usefulness hinges on recalling the right information and excluding distractors. The adaptive strategy’s precision of 0.50 and recall of 0.88 translate to fewer irrelevant passages for clinicians to sift through and a lower risk of missing details with safety implications [69,70,71,72]. Models’ performance evaluations repeatedly converged on two observations. First, responses grounded in adaptive segments were more likely to include qualifying clauses that alter action thresholds; for example, instructions to begin saline irrigations after splint removal, together with the caveat not to irrigate in the presence of active bleeding. Such clauses frequently appear in the immediately adjacent sentence and are precisely the content lost by fixed windows that split across overlaps or by cluster boundaries that privilege topical purity over narrative continuity. Second, answers based on adaptive segments exhibited a clinic-like tone, such as presenting an ordered explanation with an explicit timeline and an “unless” condition, rather than a list of disconnected facts.

These differences are clinically material. In clinical decision support, the cost of omitting a safety exception can exceed the cost of including a minor extraneous tip [73]. When a system consistently surfaces spans that contain both the recommendation and its qualifiers, it reduces ambiguity for patients and lowers the burden on clinicians to correct or annotate generated responses. The top-k efficiency gains are equally relevant. Because adaptive chunks are self-contained, the first five results more often comprise distinct, actionable units rather than near-duplicates (as seen with overlapping windows) or scattered micro-facts (as seen with proposition indexing). Each retrieval slot therefore contributes new information, increasing the likelihood that the answer addresses the entire question scope. The narrowing of variability in relevance ratings (standard deviation 0.40 with adaptive versus 0.81 with basic) suggests improved stability in what is surfaced, which in practice translates into clearer self-care guidance, explicit escalation thresholds, and fewer clarifying calls [70,71,72]. In patient-facing deployments, such as portals and mobile aftercare tools, these properties reflect the intent of the clinician team more faithfully, improving communication and confidence in the system.

It is worth noting that RAG is expanding its role from specialty-specific clinical decision support to broader public-health applications, where fast, verifiable synthesis of medical knowledge can improve triage efficiency, outbreak communication, and population-level safety messaging. In this context, enhancing retrieval fidelity and contextual completeness, as demonstrated in our adaptive chunking framework, supports not only bedside reasoning but also scalable, transparent guidance across care settings. Recent frameworks such as MedRAG and MedGraphRAG highlight how augmenting retrieval with biomedical knowledge graphs and structured reasoning can reduce hallucination rates and improve factual alignment in high-volume health information environments [74,75]. Similarly, graph-based systems like TrumorGPT extend RAG to automated fact-checking, underscoring the growing convergence between clinical decision-support reliability and public-health misinformation control [76]. Together, these advances situate our adaptive-chunking approach within a continuum of work aimed at making medical LLMs both clinically dependable and communicatively trustworthy, where retrieval precision, contextual integrity, and factual verification jointly define safety in AI-assisted healthcare.

### 4.4. Strengths and Limitations

A key strength of this work is its controlled, head-to-head design, which isolates the causal effect of segmentation by holding the knowledge base, embedding model, retriever configuration, language model, and top-k constant. Outcomes were assessed with clinician-graded accuracy and relevance alongside transparent retrieval metrics, enabling triangulation between qualitative judgment and quantitative performance. The adaptive method remained computationally practical, reusing sentence embeddings computed during pre-processing, applying a linear pass to determine boundaries, and attaching concise micro-headers with minimal overhead [50,57,68].

However, there are still several limitations. Although bootstrap resampling provided empirical confidence intervals demonstrating that results were stable across repeated draws, the evaluation set comprised thirty postoperative rhinoplasty questions, a scope appropriate for early validation but limited relative to the heterogeneity of postoperative scenarios, including late complications and revision cases, as well as alternate clinical situations concerning clinical decision support. Accuracy and relevance ratings, while clinically grounded, were subjective, and inter-rater reliability was not reported here. This decision reflected the exploratory scope of the study and limited sample size; future multi-rater validations will formally assess agreement using intraclass correlation or weighted kappa. In addition, we used a 3-point Likert scale to prioritize clarity over granularity in categorical judgments of correctness. Although appropriate for our aims, this choice may compress subtle distinctions that a 5- or 7-point scale could capture; future work should test whether added scale resolution materially improves rater discrimination or merely introduces noise. Hyperparameters, such as the number of clusters for the semantic method, the cosine similarity threshold and length budget for the adaptive method, and the policy for micro-header generation were tuned locally. These choices may require adjustment in other specialties. Overly strict similarity gates risk splitting concepts that clinicians expect to remain together, whereas permissive gates risk topic spillover. The generalizability of this approach beyond postoperative rhinoplasty remains to be established. Although the underlying retrieval and chunking mechanisms are domain-agnostic, specialty-specific terminology, documentation style, and evidence density can influence performance. Applying the framework to other surgical and non-surgical specialties will be necessary to validate robustness and to determine whether parameter adjustments or specialty-tuned indexing strategies are required.

Because the RAG knowledge base and the reference materials used for adjudication were drawn from the same authoritative sources, there is a risk of reference–corpus circularity: responses can be judged “correct” by citing the very documents that supplied the retrieved context. This setup can modestly inflate retrieval and relevance metrics and underestimate hallucination risk. We partially mitigated this by grading clinical correctness and completeness rather than textual overlap. Nonetheless, stronger bias controls will be required in future studies.

Finally, while the corpus was curated from authoritative and peer-reviewed sources to minimize bias, real-world deployment will require handling heterogeneous document quality, conflicting evidence, and near-duplicate materials. Future work should incorporate diversity constraints, contradiction detection, and enhanced source filtering before conditioning the model to ensure reliable generalization across clinical contexts.

### 4.5. Future Directions

The next step is a prospective, multisite evaluation embedded in real workflows, using prespecified endpoints that reflect clinical safety and utility. Beyond accuracy and relevance on the established three-point scales, the primary safety endpoint should be a “Safety-Clause Recall” that captures whether responses include applicable exceptions and escalation triggers, paired with operational measures such as time-to-safe-answer, callback volume, and escalation rates. A stepped-wedge or cluster-randomized design with blinded adjudication and inter-rater agreement reporting would allow each site to act as its own control while quantifying decision latency and reviewer workload under routine conditions.

Clinical deployment should proceed with a clinician-on-the-loop and explicit guardrails: page-level provenance and an always-available abstention path for underspecified or conflicting inputs; contradiction screening and near-duplicate suppression before context packing; and temporal validity checks that surface effective dates for guidance. Service targets must be defined and met, while a lightweight safety dashboard tracks Safety-Clause Recall, unsupported-citation rates, contradiction flags, abstention frequency, and drift in accuracy/relevance, with thresholds that trigger rollback to a known-good configuration. This combination of verifiable sourcing, measured safety, and predictable latency is what converts technical gains into dependable bedside tools.

Although pilot testing informed the chosen parameters, the absence of a formal ablation or sensitivity analysis remains a limitation. We did not isolate the individual contribution of micro-headers or systematically vary the similarity threshold (0.8) and span length (~500 words), as this study prioritized evaluating segmentation effects under fixed, reproducible conditions. Future work should explore how each parameter influences retrieval precision and recall through controlled ablation and threshold-sensitivity studies.

Finally, portability and equity require testing across specialties, institutions, and document styles, with attention to readability and language register so that outputs remain clear while preserving citations for clinician verification. Integration with core clinical metadata should be prioritized to tighten the retrieval scope, and updates to guideline content should follow a versioned, auditable change process. Together, these steps articulate a pragmatic path from benchmark performance to a monitored, clinician-trusted RAG-LLM that consistently delivers safe, actionable clinical guidance.

## 5. Conclusions

Our investigation demonstrates that advanced chunking, especially adaptive segmentation, substantially improves RAG-LLM performance for clinical decision support, as evidenced in the postoperative rhinoplasty setting. By aligning boundaries with clinical discourse, preserving the directive, timing, and exception within a single retrievable span, and using concise micro-headers to narrow query–document phrasing gaps, we closed a retrieval failure mode that hinders fixed windows and over-fragmented proposition splits. This design produced the most accurate, relevant, and balanced precision–recall profile among the strategies tested, yielding clinic-like answers grounded in authoritative evidence.

Although our study is confined to a well-defined, lower-risk domain, it lays the groundwork for broader deployments across specialties where guideline-based care depends on complete instruction units. By reducing reviewer burden and consistently surfacing verifiable, context-rich passages, adaptive-chunking RAG pipelines can meaningfully enhance how clinicians access and apply knowledge, ultimately supporting faster, safer, and more consistent decisions in an increasingly data-driven healthcare environment.

Looking ahead, validation should prioritize clinical risk management and hallucination suppression with explicit quantitative criteria. We propose prospective, workflow-embedded evaluations (e.g., stepped-wedge or cluster-randomized designs) with prespecified safety endpoints, including Safety-Clause Recall (proportion of responses that correctly include applicable exceptions/escalation triggers), Unsupported-Claim Rate (share of statements lacking retrieved evidence), Contradiction Flag Rate (conflicts among retrieved sources), and Time-to-Safe-Answer (latency until an evidence-grounded, clinically acceptable recommendation). Operational signals, such as callback volume and escalation rates, should be tracked alongside accuracy and relevance, with dashboard thresholds that trigger rollback to a known-good configuration. These metrics, together with portability tests across additional specialties and corpora, provide a concrete path from benchmark gains to measurable, monitored safety in deployment.

## Figures and Tables

**Table 1:** Bootstrap mean accuracy, relevance, and information retrieval with 95% CI.

Chunking Strategy	Accuracy (Mean [95% CI])	Relevance (Mean [95% CI])	IR (Mean [95% CI])
Basic RAG	1.64 [1.40–1.90]	2.60 [2.33–2.87]	Precision 0.17 [0.04–0.32]
			Recall 0.40 [0.10–0.70]
			F1 score 0.21 [0.00–0.39]
Semantic	2.04 [1.77–2.33]	2.87 [2.70–3.00]	Precision 0.33 [0.16–0.52]
			Recall 0.75 [0.50–1.00]
			F1 score 0.46 [0.19–0.64]
Proposition	2.07 [1.80–2.33]	2.80 [2.57–2.97]	Precision 0.38 [0.21–0.57]
			Recall 0.71 [0.46–0.93]
			F1 score 0.49 [0.25–0.67]
Adaptive	2.37 [2.10–2.60]	2.90 [2.73–3.00]	Precision 0.50 [0.31–0.68]
			Recall 0.87 [0.69–1.00]
			F1 score 0.63 [0.36–0.78]

## Data Availability

The datasets generated and/or analyzed during the current study are not publicly available but are available from the corresponding author on reasonable request.

## Supplementary Materials

The following supporting information can be downloaded at: https://www.mdpi.com/article/10.3390/bioengineering12111194/s1, Table S1: Failure modes across segmentation strategies.
