# Supplementary material for: Comparative Evaluation of Advanced Chunking for Retrieval-Augmented Generation in Large Language Models for Clinical Decision Support
Source: Bioengineering (Basel). 2025 Nov 1;12(11):1194. doi: 10.3390/bioengineering12111194 (PMC12649634; doi:10.3390/bioengineering12111194)
Supplement: Supplementary file 1 [file bioengineering-12-01194-s001.zip › bioengineering-3917663-supplementary.pdf]

Supplementary Table S1. Failure modes across segmentation strategies.

Case	Query / Source Excerpt	Failure Mode	Cause	Clinical Risk	Adaptive Outcome
**Fixed window split**	*Query:* "How long should I keep my head elevated and when should I call?" *Source:* "Keep head elevated at 30–45° for 48 h; resume light activity after day 3; call if vision changes or severe pain."	Omitted duration and escalation clause	Window boundary split directive/timing/safety; only first span retrieved	Incomplete post-op guidance; missing escalation trigger	Full instruction preserved; recall ↑, F_1 ↑; rated accuracy +1 category
**Semantic clustering**	*Query:* "Should I start saline rinses now?" *Source:* "Begin saline rinses unless bleeding increases; avoid NSAIDs until day 7."	Omitted "unless" clause	Topic grouping by TF-IDF/k-means ignored directive–exception dependency	Unsafe recommendation during bleeding	Directive and exception kept together; safety restored
**Proposition fragmentati on**	*Query:* "When can I blow my nose and what fever is concerning?" *Source:* "Do not blow nose for one week; if fever > 38.5 °C, call clinic."	Lost timing or misplaced fever rule	Over-granular propositions detached linkers	Missed duration or escalation threshold	Coherent span with directive + timing + safety; F_1 ↑
**Adaptive edge case**	*Source:* "Change tape daily… Until day 10, avoid pressure on nasal dorsum."	Minor timing mislink	Timing cue in another section	Slight under-linking of temporal scope	Boundary repair and overlap reduced issue; future fix: cross-section linking
